# Global prevalence of developmental disabilities in children and adolescents: A systematic umbrella review

**DOI:** 10.3389/fpubh.2023.1122009

**Published:** 2023-02-16

**Authors:** Bolajoko O. Olusanya, Tracey Smythe, Felix A. Ogbo, M. K. C. Nair, Mark Scher, Adrian C. Davis

**Affiliations:** ^1^Centre for Healthy Start Initiative, Lagos, Nigeria; ^2^International Centre for Evidence in Disability, London School of Hygiene & Tropical Medicine, London, United Kingdom; ^3^Division of Physiotherapy, Department of Health and Rehabilitation Sciences, Stellenbosch University, Cape Town, South Africa; ^4^Riverland Academy of Clinical Excellence (RACE), Riverland Mallee Coorong Local Health Network, SA Health | Government of South Australia, Berri, SA, Australia; ^5^Translational Health Research Institute (THRI), Campbelltown Campus, Western Sydney University, Penrith, NSW, Australia; ^6^NIMS-Spectrum-Child Development Research Centre, NIMS Medicity, Thiruvananthapuram, Kerala, India; ^7^Department of Pediatrics, Division of Pediatric Neurology, Fetal-Neonatal Neurology Program, Rainbow Babies and Children's Hospital, University Hospitals Cleveland Medical Center, Cleveland, OH, United States; ^8^MacDonald Hospital for Women, Case Western Reserve University School of Medicine, University Hospitals Cleveland Medical Center, Cleveland, OH, United States; ^9^Department of Population Health, London School of Economics, London, United Kingdom; ^10^Vision and Eye Research Institute, School of Medicine Anglia Ruskin University, Cambridge, United Kingdom

**Keywords:** developmental disabilities, global health, Global Burden of Disease, developmental epidemiology, early childhood development, inclusive education, SDG 4.2

## Abstract

**Aim:**

The provisions of the United Nation's Sustainable Development Goals (SDGs) for disability-inclusive education have stimulated a growing interest in ascertaining the prevalence of children with developmental disabilities globally. We aimed to systematically summarize the prevalence estimates of developmental disabilities in children and adolescents reported in systematic reviews and meta-analyses.

**Methods:**

For this umbrella review we searched PubMed, Scopus, Embase, PsycINFO, and Cochrane Library for systematic reviews published in English between September 2015 and August 2022. Two reviewers independently assessed study eligibility, extracted the data, and assessed risk of bias. We reported the proportion of the global prevalence estimates attributed to country income levels for specific developmental disabilities. Prevalence estimates for the selected disabilities were compared with those reported in the Global Burden of Disease (GBD) Study 2019.

**Results:**

Based on our inclusion criteria, 10 systematic reviews reporting prevalence estimates for attention-deficit/hyperactivity disorder, autism spectrum disorder, cerebral palsy, developmental intellectual disability, epilepsy, hearing loss, vision loss and developmental dyslexia were selected from 3,456 identified articles. Global prevalence estimates were derived from cohorts in high-income countries in all cases except epilepsy and were calculated from nine to 56 countries. Sensory impairments were the most prevalent disabilities (approximately 13%) and cerebral palsy was the least prevalent disability (approximately 0.2–0.3%) based on the eligible reviews. Pooled estimates for geographical regions were available for vision loss and developmental dyslexia. All studies had a moderate to high risk of bias. GBD prevalence estimates were lower for all disabilities except cerebral palsy and intellectual disability.

**Conclusion:**

Available estimates from systematic reviews and meta-analyses do not provide representative evidence on the global and regional prevalence of developmental disabilities among children and adolescents due to limited geographical coverage and substantial heterogeneity in methodology across studies. Population-based data for all regions using other approaches such as reported in the GBD Study are warranted to inform global health policy and intervention.

## Introduction

The United Nations' Sustainable Development Goals (SDGs) are widely embraced, especially in low- and middle-income countries (LMICs), as the priority global agenda for improving population health and well-being by 2030 (1). The disability-inclusive provisions of the SDGs have stimulated a growing interest in children and adolescents (hereinafter reported as “children”) with developmental disabilities globally (2, 3). The Convention on the Rights of Persons with Disabilities (CRPD) defines persons with disabilities to include “those who have long-term physical, mental, intellectual or sensory impairments which in interaction with various barriers may hinder their full and effective participation in society on an equal basis with others” (4). Developmental disabilities are frequently defined as chronic physical, cognitive, speech or language, psychological, or self-care conditions that typically originate during childhood before the age of 22 years; are likely to continue indefinitely; and require additional coordinated services, support, or other assistance for an extended duration or during a lifetime; and represent a subset of conditions that affect children with special health care needs (5, 6). Right from birth, children with developmental disabilities, especially in LMICs experience stigma along with negative attitudes and beliefs that place them at increased risk of neglect, exploitation, and violence, as well as premature death including infanticide (2). These children also perform significantly poorer than children without disabilities across virtually all indicators of health and educational wellbeing in early childhood (2).

Up-to-date prevalence estimates are essential to raise awareness and inform policy initiatives, service planning, resource allocation, and research priorities (2). Evidence from global health databases suggests that about 240 million children globally have developmental disabilities based on parent-reported functional difficulties compared to 290 million children using statistical modeling techniques (3). Although systematic reviews and meta-analyses are more suited for evaluating the effectiveness of health interventions and accuracy of diagnostic tests from clinical trials (7–9), it is not uncommon to use pooled prevalence estimates from individual primary studies as proxies for the global and regional prevalence of children with developmental disabilities (10–13). However, it is unclear how such prevalence estimates compare with those reported in global health databases from the World Health Organization (WHO), United Nations Children's Fund (UNICEF), the World Bank or the Global Burden of Disease (GBD) Study published by the Institute for Health Metrics and Evaluation (IHME), USA. Umbrella reviews are increasingly being used to summarize evidence from systematic reviews and meta-analyses, especially for health care interventions (14, 15). We, therefore, set out to conduct an umbrella review of systematic reviews and meta-analyses of the prevalence estimates of developmental disabilities for comparison with estimates from other sources of population data in global health. The primary goal of this umbrella review was to provide a narrative synthesis of the selected reviews due to well-documented differences in the methodological approaches to disability measurement (3).

## Methods

The protocol for this systematic umbrella review was registered in the International Prospective Register of Systematic Reviews (PROSPERO), reference number #CRD42022373552 (https://www.crd.york.ac.uk/prospero/#searchadvanced). We adopted the Preferred Reporting Items for Overviews of Reviews (PRIOR) statement for conducting umbrella reviews (16). This statement was considered more up-to-date and better suited for an umbrella review than the Preferred Reporting Items for Systematic Reviews and Meta-Analyses (PRISMA). The term “reviews” in this paper is used for published articles that are systematic reviews and meta-analyses of primary studies. The term “primary studies” refers to any original research or investigation conducted to determine the prevalence of specific developmental disabilities in a defined population.

### Search strategy and selection criteria

We searched PubMed, Scopus, EMBASE, PsycINFO, and Cochrane Library in October 2022 using the terms (“prevalence” OR “incidence”) AND (“disability” OR “impairment” OR “disorder”), filtered for systematic reviews and meta-analyses, English Language, and children under 20 years published between September 2015 (when the SDGs were launched) and August 2022. Eligible systematic reviews were those that were peer-reviewed with a clearly stated research question, systematic search of at least two databases and systematic data synthesis. No supplementary search for primary studies was conducted (16). The GBD Study from IHME (https://vizhub.healthdata.org/gbd-results/) is presently the only global health database that provides global, regional, and national prevalence estimates of specific disabilities among children and adolescents according to the American Psychiatric Association's (APA's) Diagnostic and Statistical Manual of Mental Disorders (DSM) (17), or WHO's International Classification of Diseases (ICD) codes (18). The selection of specific disabilities for our umbrella review was therefore guided by those typically reported by GBD database to facilitate appropriate comparability (3). These disabilities include attention-deficit/hyperactivity disorder (ADHD), autism spectrum disorder (or simply “autism” hereinafter), cerebral palsy, developmental intellectual disability, epilepsy, hearing loss and vision loss. We also included developmental dyslexia because of its relevance to the disability-inclusive education provision in the SDGs (1). Developmental dyslexia is a specific impairment characterized by severe and persistent problems in the acquisition of reading skills and it is not typically reported by GBD. Two independent reviewers/authors (BOO and TS) searched titles and abstracts for eligibility and evaluated the full texts of the eligible articles for inclusion. Any unresolved conflict was to be referred to a third reviewer/author (FAO) for adjudication. Reviews that provided pooled estimates with confidence intervals of the selected disabilities were included. In general, these reviews assessed the heterogeneity of the eligible primary studies and performed random effects meta-analysis to estimate the pooled prevalence of a disability. No distinction was made between reviews that evaluated population-based primary studies and those based on a random sample of participants. We excluded reviews that focused on a specific population group such as children who are born preterm, those with different birth weights, refugees, children exposed to HIV or malnourished children. We also excluded reviews that reported a subset of children with a specific disability such as children with refractive errors among those with vision loss as well as reviews that were published before September 2015, that focused on specific countries, one geographical region, or had less than 10 primary studies as such reviews were unlikely to accurately reflect the overall prevalence of disability among all children and adolescents. In order to minimize the risk of missing other relevant systematic reviews, a further manual search of PubMed and selected child health journals was conducted specifically for each of the eight selected disabilities. The reference lists of included reviews were also searched for the identification of additional eligible references.

### Data extraction

The citations for the retrieved reviews were first migrated to separate spreadsheets based on the standard fields in each database. A combined spreadsheet was then created for the selected articles with the following fields: source database, year of publication, authors, title, journal, abstract and journal link to the full text. From the full text of the selected articles, the following information were extracted by two authors (BOO and TS): name of disability, citation, year of publication, databases searched, number of primary studies, number of countries covered, proportion of countries from LMICs, overall study size, age group of the reported prevalence estimate, global prevalence estimate, prevalence estimate for high-income countries (HICs), prevalence estimate for LMICs, and remarks. The composition of HICs and LMICs is based on the 2022 World Bank classification (https://data.worldbank.org/country/XO).

### Evaluation of the methodological quality

The risk of bias (quality) of included reviews was assessed independently by two reviewers (BOO and TS). The Assessment of Multiple Systematic Reviews (AMSTAR2) tool (available at https://amstar.ca/Amstar-2.php) (19) and the Joanna Briggs Institute (JBI) Critical Appraisal Checklist for umbrella reviews (20) were used as neither tool covered all relevant sources of bias in reviews on the prevalence estimates of developmental disabilities. For instance, AMSTAR2 was specifically designed for health intervention research but it is more comprehensive than JBI checklist and accounts for the quality of the primary studies included in the meta-analysis, without limiting the quality assessment to the technical aspects of the meta-analysis itself. The AMSTAR2 questionnaire has 16 criteria and requires reviewers to respond with a “Yes” or “Partial Yes” or “No” or “No Meta-analysis” option. Overall quality was classified as “critically low,” “low,” “moderate,” and “high” (17). JBI consists of 10 criteria scored as being “met” (1), “not met” (0), or “unclear” (UC), resulting in an overall quality score of 0 to 10. The scores were categorized as low (0–4), medium (5–7), and high-quality (8–10) reviews. Disagreements on risk of bias ratings were resolved through discussion.

### Global Burden of Disease estimates

The latest GBD estimates of developmental disabilities in children and adolescents in 2019 were obtained from two publications (3, 21), which were extracted from the substantive GBD 2019 Database (https://vizhub.healthdata.org/gbd-results/) and the GBD-WHO Rehabilitation Database or “WHO Rehabilitation Need Estimator” (https://vizhub.healthdata.org/rehabilitation/). These are the only sources of global and regional prevalence estimates of specific developmental disabilities covering 204 countries and territories, including the 193 UN Member States. The GBD methodology has been extensively reported (3, 21, 22). In summary, the prevalence estimation for each condition begins with the compilation of all available data inputs from systematic reviews of the literature, hospital and claims databases, health surveys, case notification systems, cohort studies, and multinational survey data. A comprehensive list of the sources of input data for each condition is publicly available at the Global Health Data Exchange (https://ghdx.healthdata.org/gbd-2019/data-input-sources). In the data preparation, efforts were made to (i) optimize the comparability of data derived from various sources using different methods; (ii) find a consistent set of estimates across prevalence data; and (iii) generate estimates for locations with sparse or no data by using available information from other locations combined with covariates. Prevalence estimates are then generated using DisMod-MR 2.1, a statistical modeling technique developed specifically for the GBD project. This is a Bayesian meta-regression tool that synthesizes epidemiological data for fatal and non-fatal health outcomes from disparate settings and sources, adjusting for different case definitions/diagnostic criteria or sampling methods, to generate internally consistent estimates by geographical location, year, age group, and sex. The GBD database contains estimates from 1990 to 2019 and are accompanied by the corresponding 95% uncertainty bounds intervals (UI). Prevalence estimates are available for seven of the eight selected disabilities. Developmental dyslexia is presently not included in the GBD databases. We did a narrative synthesis of included studies in comparison to the GBD (2019) study and compared prevalence estimates for the eight selected disabilities.

## Results

**Figure 1:**
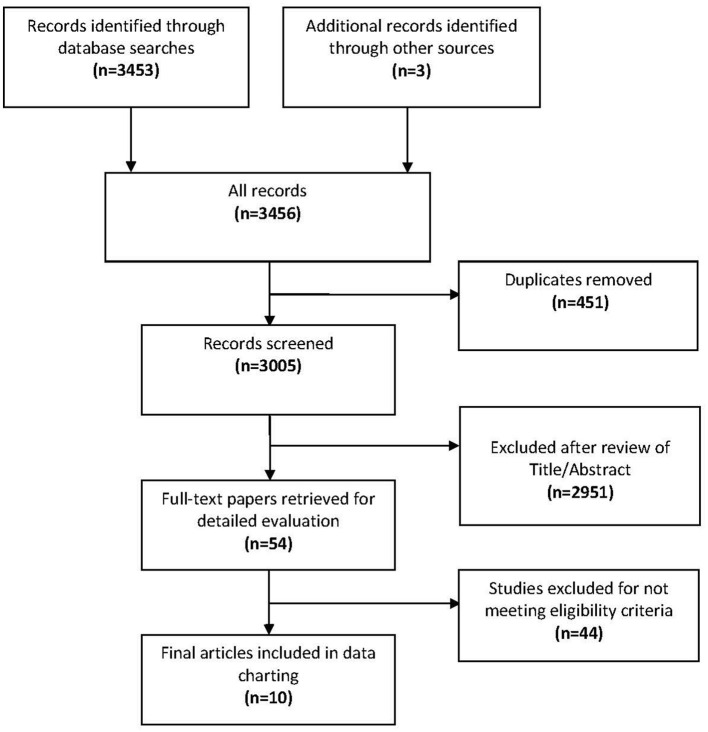
Flow diagram of the study selection process and results.

**Figure 2:**
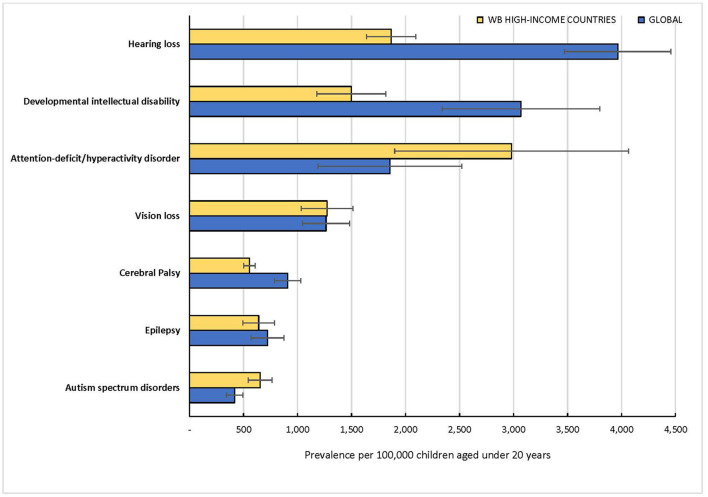
Prevalence estimates of selected developmental disabilities in children under 20 years in 2019 by the Global Burden of Disease (GBD) Study Group.

The initial search of the five bibliographic databases yielded 3453 articles composed as follows: Scopus (*n* = 1,788), PubMed (*n* = 681), EMBASE (*n* = 755), PsycINFO (*n* = 87) and Cochrane Library (*n* = 142). Three articles were identified from outside the databases giving a total of 3,456 articles (Figure 1). A total of 54 articles were selected for full-text review based on the inclusion and exclusion criteria. After the review of the full-texts, 44 articles were excluded and the reasons for their exclusion are summarized in Supplementary Table S1. The most common reason for exclusion was the absence of global and regional prevalence estimates for children and adolescents. Of the 10 articles selected for inclusion that reported pooled global prevalence estimates of disabilities (10–12, 23–29), three articles focused on ASD and the remaining seven articles were each focused on one disability. A summary of the selected reviews is presented in Table 1. The primary studies covered by the selected systematic reviews and meta-analyses ranged from 14 to 88 articles and the vast majority were from HICs. The reported age groups varied across most reviews except for cerebral palsy, hearing loss and vision loss. Prevalence estimates of developmental disabilities in LMICs were only reported for ASD, cerebral palsy, and developmental dyslexia. Prevalence estimates for the WHO or World Bank world regions were reported for developmental dyslexia and vision loss. Since the prevalence estimates from most of the systematic reviews were derived from primary studies conducted in HICs, the GBD global estimates were reported along with the estimates for HICs as prevalence estimates for LMICs as a group are not reported separately by GBD (Figure 2).

**Table 1:** Prevalence estimates of developmental disabilities in children and adolescents reported in systematic reviews (2015–2022) compared to GBD 2019 estimates.

**Condition**	**N**	**References**	**Year of publication**	**Databases used**	**Number studies**	**Countries [LMICs]**	**Overall study size**	**Age group**	**Prevalence_Global (95% confidence interval)^#^**	**Prevalence [HICs]**	**Prevalence [LMICs]**	**Remarks**	**GBD 2019 [0–19 years]**
Attention-deficit/hyperactivity disorder (ADHD)	1	Barican et al. (23)	2022	MEDLINE, EMBASE	14	11 [0]	61,545	4–18 years	3.7% (2.3–5.7)	3.7% (2.3–5.7)	Not reported	Regional population of children with condition.	1.9% (1.3–2.6)
Autism spectrum disorder (ASD)	2	Zeidan et al. (11)	2022	MEDLINE	71	34 [16]	Not reported	0–89 years, predominantly below 18 years	100/10,000 (IQR: 1.09/10,000 to 436.0/10,000)	Not reported	Not reported	Regional population of children with condition not reported.	0.4% (0.3–0.5)
	3	Salari et al. (25)	2022	Science Direct, PubMed, Scopus, SID, Magiran, Web of Science, Google Scholar	74	41 [15]	30,212,757	0–27 years	0.6% (0.4–1)	Not reported	Not reported	Limited regional population of children with condition reported.	
	4	Wang et al. (26)	2022	PubMed, EMBASE, Web of Science	51	25 [6]	548,413,748	All ages, predominantly school children	98/10,000 (81/10,000–118/10,000)	85/10,000 (67/10,000–105/10,000)	155/10,000 (111/10,000–204/10,000)	Limited regional population of children with condition reported.	
Cerebral palsy	5	McIntyre et al. (10)	2022	MEDLINE, EMBASE	41	27 [6]	Not reported	0–18 years	Not reported	1.6/1,000 (1.5–1.7) live births	3.4/1,000 (3.0–3.9) live births	Global and regional population of children with condition not reported.	0.9% (0.8–1.0)^#^
Developmental intellectual disability (DID)	6	McKenzie et al. (27)	2016	PubMed, MEDLINE, EMBASE, PsycINFO, Cochrane	18	9 [2]	Not reported	Child & adolescent	0.22–1.55%	Not reported	Not reported	Pooled estimate not reported. Highest reported estimate came from USA in 1996.	3.1% (2.3–3.8)
Epilepsy	7	Fiest et al. (28)	2017	MEDLINE, EMBASE	24	42 [34]	Not reported	0–9 years 10–19 years	5.19/1,000 (3.54–7.62) [0–9 years]; 8.86/1,000 (6.58–11.92) [10–19 years]	Not reported	Not reported	Regional population of children with condition not reported.	0.7% (0.6–0.9)
Hearing loss	8	Wang et al. (29)	2019	MEDLINE, EMBASE	88	39 [23]	3,360,850	0–18 years	13.1% (10.0–17.0) [>15 dBHL]; 8.1% (1.3–19.8) [>20 dBHL]	Not reported	Not reported	Global and regional population of children with condition not reported.	4.0% (3.5–4.5)
Vision loss	9	Yekta et al. (12)	2022	PubMed, Scopus, and Web of Science	80	28 [19]	769,720	0–19 years	12.72% (9.26–16.19) [UCVA of 20/40 or worse in better eye]; 7.26% (4.34–10.19) [UCVA of 20/60 or worse in better eye]	Not reported	Not reported	Global and regional population of children with condition reported.	1.3% (1.1–1.5)
Developmental dyslexia	10	Yang et al. (30)	2022	PubMed, EMBASE, Web of Science, Cochrane, EBSCO host, ProQuest, Springerlink, & 5 Others^∧^	58	16 [6]	Not reported	6–13 years	7.10% (6.27–7.97)	7.10% (5.54–8.82)	7.10% (6.10–8.20) [MICs]	Global and regional population of children with condition reported.	Not Available

GBD, Global Burden of Disease (GBD); LMICs, Low- and middle-income countries; MICs, Middle-income countries; HICs, High-income countries; UCVA, Uncorrected visual acuity.

* Except stated otherwise,

# Derived from GBD-WHO Rehabilitation Need Estimator Database,

∧ China National Knowledge Infrastructure, Wanfang, CQ-VIP, China Hospital Knowledge Database, OATD database.

### Attention-deficit/hyperactivity disorder

Barican et al. reported a pooled prevalence of 3.7% (95% CI: 2.3–5.7) in children aged 4–18 years from 14 primary studies in 11 countries (23). The primary studies covered the period January 1990 to February 2021, and specifically excluded studies from LMICs. The GBD estimated the prevalence of ADHD among children 0–19 years as 1.9% (95% UI: 1.3–2.6) in 2019 (Table 1). The GBD prevalence estimate of ADHD for HICs is approximately 3.0% (95% UI: 2.0–4.2), suggesting a far lower estimate for LMICs (Figure 2).

### Autism spectrum disorder

Three reviews all published in 2022 reported prevalence estimates of ASD DHD that ranged from 0.6% (95% CI: 0.4–1.0) to a median of 1.0% (Interquartile range: 1.1–4.4) (11, 24, 25). One study by Barican et al. reported estimates for ADHD and ASD, but the estimate for ASD was not considered as it was derived from only four primary studies (23). None of the reviews provided pooled estimates specifically for children and adolescents. The primary studies covered ranged from 51 to 71 articles derived from 25 to 41 countries, less than half of which were LMICs in all three reviews. One of the reviews by Wang et al. aimed to determine the prevalence of gastrointestinal symptoms in individuals with ASD and reported pooled estimates of ASD for HICs (0.9%, 95% CI: 0.8–1.2) and LMICs (1.6%, 95% CI: 1.1–2.0) (25). Four of the 51 primary studies in this review involved individuals older 20 years or older and the selected studies were published between 2001 and 2022. Regional estimates were reported for Africa (3.0%, 95% CI: 2.5–3.4), Oceania (2.6%, 95% CI: 1.6–3.8), the Americas (1.3%, 95% CI: 1.1–1.6), Asia (0.3%, 95% CI: 0.3–0.4) and Europe (0.7%, 95% CI: 0.6–0.8). The GBD global estimate for ASD was 0.4% (95% UI: 0.3–0.5) with a higher prevalence of 0.7% (95% UI: 0.6–0.8) estimated for HICs (Figure 2), suggesting a lower prevalence for LMICs compared to HICs.

### Cerebral palsy

The included systematic review by McIntyre et al. reported prevalence estimates for HICs and LMICs separately (10). A total of 41 primary studies were included in the review derived predominantly from surveillance registries in 27 countries, six of which were LMICs. The review covered studies published between January 2011 and November 2020 and the sample included children with birth year of 1995 and beyond. The estimated birth prevalence of cerebral palsy was approximately 0.2% (95% CI: 0.1–0.2) for HICs and 0.3% (95% CI: 0.3–0.4) for LMICs among children 0–18 years. A pooled global estimate was not reported nor estimates by geographical world regions. The meta-analysis was based on children with birth year from 2010. The GBD estimate for cerebral palsy was 0.9% (95% UI: 0.8–2.0) globally based on children with moderate to severe motor impairment (21). The prevalence estimate for HICs was 0.6% (95% UI: 0.5–0.6) which would suggest a higher prevalence for LMICs than the reported global estimate.

### Developmental intellectual disability

Only one systematic review by McKenzie et al. published in 2016 was identified for this study (26). The review included primary studies published between 2010 to 2015 and no meta-analysis was conducted. There were 18 primary studies covering all age groups from 9 countries, and all but 2 countries were HICs. Prevalence was highly variable across studies and ranged from 0.22 % in 2007–2008 (USA) to 1.55 % in 1996 (USA) among children and adolescents. The GBD global estimate was 3.1% (95% UI: 2.3–3.8) and the estimate for HICs was 1.5% (95% UI: 1.2–1.8), suggesting a significantly higher prevalence for LMICs than the global estimate (Figure 2).

### Epilepsy

One systematic review by Fiest et al. published in 2017 was eligible for inclusion (27). The review covered the period from 1985 to October 2013 and included 63 primary studies in all age groups (0–60+ years) from 42 countries, only 8 of which were HICs. Prevalence estimate was reported separately for children aged 0–9 years (0.5%, 95% CI: 0.4–0.8) and children/adolescents aged 10–19 years (0.9%, 95% CI: 0.7–1.2). Overall pooled estimates for all age groups were reported separately for HICs and LMICs but not for children and adolescents. The GBD global estimate was 0.7% (95% UI: 0.6–0.9) and the estimate for HICs was 0.6% (95% UI: 0.5–0.8), suggesting a significantly higher prevalence for LMICs than the global estimate (Figure 2).

### Hearing loss

The systematic review by Wang et al. published in 2019 was the only eligible study (28). The review was specifically conducted for children aged 0–18 years and included 88 articles published between January 1996 and August 2017 from 39 countries, 23 (or roughly 60%) of which were LMICs. The review computed pooled estimates at different hearing threshold levels, and the prevalence decreased as the severity of hearing loss (the threshold cutoff) increased. Prevalence estimates ranged widely from as low as 0.1% (95% CI, 0.1–0.2) when hearing loss was defined using a lower frequency average (0.5, 1, and 2 kHz) with a hearing threshold/level of 40 decibel (40-dBHL) in both ears to as high as 17.9% (95% CI: 15.9–20.0) when using a full frequency average (0.5 to 8 kHz) with a 15 dBHL threshold in 1 or both ears. Two global prevalence estimates using the most reported thresholds for hearing loss were presented: 13.1% (95% CI: 10.0–17.0) based on >15 dBHL and 8.1% (95% CI: 1.3–19.8) based on >20 dBHL. As recommended by the WHO, the GBD uses 20 dBHL threshold for all its computations. The global prevalence was estimated as 4.0% (95% UI: 3.5–4.5) while the estimate for HICs was 1.9% (95% UI: 1.6–2.1), which suggests a higher prevalence for LMICs than the global estimate (Figure 2).

### Vision loss

One systematic review by Yekta et al. published in 2022 met our inclusion criteria (12). The review included 80 studies published between 1971 and 2018 from 28 countries, 19 of which are LMICs. It is the only systematic review that was specifically conducted among children and adolescents below 20 years. It was also the only review that reported estimates for all WHO regions. The global prevalence of vision loss was 12.7% (95% CI: 9.3–16.2) based on uncorrected visual acuity (UCVA) of 20/40 or worse in the better eye, and 7.3% (95% CI: 4.3–10.2%) based on UCVA of 20/60 or worse in the better eye. The GBD global prevalence was estimated as 1.3% (95% UI: 1.1–1.5) using visual acuity of less than 6/18 according to the Snellen chart, while the estimate for HICs was 1.3% (95% UI: 1.1–1.5) (Figure 2).

### Developmental dyslexia

One systematic review by Yang et al. published in 2022 provided the most comprehensive and up-to-date status of children with developmental dyslexia globally (29). The review covered 58 primary studies published as far back as the 1950s until June 2021 and involved school children aged 6–13 years. A total of 58 studies were selected for the review drawn from 16 countries, 6 of which were LMICs. The pooled global prevalence was 7.1% (95% CI: 6.3–8.0%). The prevalence estimates for HICs (7.1%, 95% CI: 5.5–8.8%) and middle-income countries (7.1%, 95% CI: 6.1–8.2%) were similar. Pooled estimates based on WHO regions were also reported. However, developmental dyslexia is not included in the GBD database.

### Risk of bias

The quality of the selected reviews is summarized in Supplementary Tables S2, S3. The inter-rater reliability after the first round of independent evaluation was 94.8% for the AMSTAR2 and 98.3% for JBI Checklist. Differences were resolved by consensus. For example, the AMSTAR2 required authors to provide a list of excluded reviews and justify the exclusions. This accounted for most of the discrepancies between the two raters. It was therefore agreed that reviews that reported the excluded primary studies in the PRISMA flow diagram with explanations for the exclusion should be considered as satisfying this criterion. Based on AMSTAR2, none of the reviews met the criteria for high quality and the most were either of low or critically low quality. In contrast, based on JBI checklist, none of the reviews were of low quality. In fact, 9 of the reviews were of high quality and 3 of medium quality.

## Discussion

We set out to provide an overview of the pooled prevalence estimates of commonly reported disabilities in children and adolescents derived from systematic reviews and meta-analyses, published approximately midway into the SDGs and to compare the findings with estimates from alternative data sources in global health. To our best knowledge, this is the first systematic umbrella review on the global prevalence of the selected disabilities in children and adolescents. The principal finding was that sensory impairments were the most prevalent disabilities (13.1% for hearing loss and 12.7% for vision loss) while cerebral palsy was the least prevalent disability (approximately 0.2%) globally.

Another important finding was that most of the global prevalence estimates were derived from primary studies conducted in HICs and estimates for LMICs were reported for only three disabilities: autism spectrum disorder, cerebral palsy, and developmental dyslexia. The highest number of countries providing primary data for any disability was 56, which is 29% of all UN Member States that signed the SDGs. Regional prevalence estimates were only available for autism spectrum disorder, vision loss and developmental dyslexia. In contrast, the GBD estimates were available for high-income countries which gave indications on the contribution of LMICs to the global prevalence for the selected disabilities except developmental dyslexia. For example, the contributions of LMICs to the global prevalence of hearing loss and intellectual disability were substantially higher than those from high-income countries, in contrast to findings on autism spectrum disorder and ADHD.

Another notable finding was that the age groups of children reported in the reviews varied which makes direct comparison of estimates challenging. Furthermore, the global prevalence estimates reported for ADHD, autism, epilepsy, hearing loss and vision loss in systematic reviews were higher than those reported by the GBD. In contrast, prevalence estimates for cerebral palsy and intellectual disability from systematic reviews were lower than those reported by GBD. Based on GBD data, hearing loss was the most prevalent disability (4.0%) and autism was the least prevalent (0.4%) disability in children and adolescents. The modeling techniques used by GBD for each of the disabilities and the number of countries covered would have accounted for the differences in the global prevalence estimates between the GBD and the systematic reviews. However, the pooled prevalence estimate for cerebral palsy for LMICs of approximately 0.3% does not appear to reflect the well documented disproportionately high burden of the risk factors for cerebral palsy and the reported prevalence estimates in young children in LMICs, especially in South Asia and sub-Saharan Africa (21, 30, 31). For example, in one robust population-based study in India, the prevalence estimate of up to 2.1% for neuromotor impairments including cerebral palsy was reported (31).

Another major finding was the sharp contrast in the quality rating of the included reviews from two different assessment tools. The major reason for the poor quality rating based on AMSTAR2 were that most of the reviews (8 out of 12) did not provide an explicit statement that the review methods were established prior to the conduct of the review which constitutes a major risk of bias in all included reviews (19). In addition, none of the reviews reported the sources of funding for the primary studies that were selected. For these and other reasons we concluded that the available reviews are generally not of a high quality to inform policy interventions in global health.

These findings would suggest that prevalence estimates derived from systematic review and meta-analyses are unlikely to provide comparable data for different disabilities to satisfy the requirements for policy and investment decisions in global health, especially in relation to population-level information for service planning. Prevalence estimates for geographical world regions were not available for most disabilities. More crucially, it was difficult to combine the estimates from the various reviews to determine an overall global estimate of disabilities in children and adolescents due to marked variability of study designs, methodological approaches, sampling strategies, and the diagnostic criteria used in case ascertainment (32). These limitations have accounted for the growing reliance by policymakers on alternative approaches and sources of global estimation of population health metrics including household surveys and statistical modeling (3, 22, 33). In order to address these limitations, the GBD for example, utilizes sophisticated statistical techniques to (i) optimize the comparability of data derived from various sources using different methods; (ii) find a consistent set of estimates across prevalence data; and (iii) generate estimates for locations with sparse or no data by using available information from other locations combined with covariates (22). However, it is important to clarify that GBD estimates are equally associated with several limitations which have been reported extensively in the literature (3, 21, 22). For example, The GBD methodology of estimating the prevalence of disabilities based on sequelae of the underlying health conditions or surrogates may result in over-estimation or under-estimation due to the difficulty in accurately accounting for idiopathic impairments. Behavioral conditions such as ASD and ADHD, continue to rely on sparse data in many regions, particularly LMICs. In addition, The GBD estimates for disabilities still do not fully reflect the complex and dynamic relationship between health conditions and contextual personal or environmental factors under the ICF, as such they provide a limited picture of disability. It is also important to mention that while cerebral palsy is least prevalent among the selected developmental disabilities, it is the leading cause of early-onset physical disability. Considering that cerebral palsy is lifelong and very disabling for some people, the impact in terms of disability-adjusted life years makes cerebral palsy a more significant condition from a public health perspective than its low prevalence might suggest (34). The use of live births as denominator in computing the prevalence, is also unlikely to reflect the extent of the disability in the population optimally.

A major strength of this study is that the findings from the systematic reviews were compared with the latest prevalence estimates in the GBD database, which is novel. We had previously demonstrated that the prevalence estimate of disabilities in children and adolescents (<20 years) by GBD and UNICEF were not statistically different and were statistically equivalent (3). The study also complied with the key quality measures recommended by AMSTAR2, including prior registration with PROSPERO and the provision of a separate list of excluded reviews and reasons for exclusion. Another unique feature was the quality evaluation of the included reviews using two separate risk-of-bias tools. We also included developmental dyslexia which is the most common type of learning disability, accounting for approximately 80% of all learning disabilities but rarely reported in the global health literature (29, 35).

A few limitations of this umbrella review are worth restating. First, the electronic databases searched were not exhaustive which would have resulted in a potential selection bias. For example, we excluded non-English articles, and we did not search Web of Science, Google Scholar, and regional databases such as the WHO Library (WHOLIS), LILACS (formerly Latin America Index Medicus) and African Index Medicus for additional eligible articles from LMICs, which could have biased the findings. Second, no meta-analysis of the reported estimates was undertaken, primarily due to heterogeneity in the methods, age groups and the sample sizes of the included reviews. However, umbrella reviews in general are aimed at summarizing the evidence rather than to re-synthesize primary studies (20). Third, there was wide variation in the period covered by selected reviews which would have made comparison of reported estimates across disabilities biased and inconsistent. Fourth, prevalence estimates reported for HICs frequently mask the health and social inequalities in rural and isolated areas designated as medical deserts due to inadequate access to medical care.

## Conclusion

Up-to-date prevalence estimates of disabilities in children and adolescents are essential to raise awareness and inform policy initiatives, service planning, resource allocation, and research priorities. However, available estimates from systematic reviews and meta-analyses do not provide representative evidence on the global and regional prevalence of developmental disabilities due to limited geographical coverage and substantial heterogeneity in methodology across the primary studies. Population-based data for all regions that reflect and adjust for these limitations such as those reported by GBD Study periodically are warranted to inform global health policy and intervention.

## Author contributions

BO drafted the manuscript. TS, FO, MN, MS, and AD critically reviewed the draft and suggested essential edits. All authors contributed to revising the manuscript and have approved of the final version.
