# Supplementary material for: Global prevalence of developmental disabilities in children and adolescents: A systematic umbrella review
Source: Front Public Health. 2023 Feb 16;11:1122009. doi: 10.3389/fpubh.2023.1122009 (PMC9987263; doi:10.3389/fpubh.2023.1122009)
Supplement: Supplementary file 1 [file Data_Sheet_1.PDF]

Supplementary Table S1. Studies Excluded with Reasons after Full-Text Review

	Excluded Studies	Reasons
**1**	Francés L, Quintero J, Fernández A, et al. Current state of knowledge on the prevalence of neurodevelopmental disorders in childhood according to the DSM-5: a systematic review in accordance with the PRISMA criteria. Child Adolesc Psychiatry Ment Health. 2022 Mar 31;16(1):27.	Selection bias. Less than 10 primary studies on ASD and ADHD reviewed
**2**	Heady N, Watkins A, John A, Hutchings H. Prevalence of neurodevelopmental disorders and their impact on the health and social well-being among looked after children (LAC): a systematic review protocol. Syst Rev. 2022 Mar 19;11(1):49.	Global or regional prevalence of children with ASD and ADHD not estimated
**3**	Kahathuduwa CN, Dhanasekara CS, Wakefield S, Moustaid-Moussa N, Mastergeorge A. Autism spectrum disorder is associated with an increased risk of development of underweight in children and adolescents: A systematic review and meta-analysis. Research in Autism Spectrum Disorders 2022; 94: 101969.	Global or regional prevalence of children with ASD not estimated
**4**	Barros A, Mascarenhas P, Botelho J, Machado V, Balixa G, Bandeira Lopes L. Autism Spectrum Disorders and Malocclusions: Systematic Review and Meta-Analyses. J Clin Med. 2022 May 11;11(10):2727.	Global and regional prevalence estimate of ASD in children not estimated
**5**	Fang Z, Cerna-Turoff I, Zhang C, Lu M, Lachman JM, Barlow J. Global estimates of violence against children with disabilities: an updated systematic review and meta-analysis. Lancet Child Adolesc Health. 2022 May;6(5):313-323.	Global and regional prevalence of disabilities in children not estimated.
**6**	Heydarian S, Abbasabadi MM, Khabazkhoob M, Hoseini-Yazdi H, Gharib M. Vision Abnormalities in Children and Young Adults With Cerebral Palsy; A Systematic Review. Semin Ophthalmol. 2022 May 19;37(4):471-479.	Global and regional prevalence of disabilities in children not estimated.
**7**	Liu X, Sun X, Sun C, et al. Prevalence of epilepsy in autism spectrum disorders: A systematic review and meta-analysis. Autism. 2022 Jan;26(1):33-50..	Review limited to prevalence of epilepsy in children with ASD
**8**	Solmi M, Radua J, Olivola M, et al. Age at onset of mental disorders worldwide: large-scale meta-analysis of 192 epidemiological studies. Mol Psychiatry. 2022 Jan;27(1):281-295.	Global and regional prevalence of ASD and ADHD in children not estimated.
**9**	GBD 2019 Mental Disorders Collaborators. Global, regional, and national burden of 12 mental disorders in 204 countries and territories, 1990-2019: a systematic analysis for the Global Burden of Disease Study 2019. Lancet Psychiatry. 2022 Feb;9(2):137-150.	Prevalence estimates for ASD, ADHD and Intellectual Disability not reported for all children < 20 years
**10**	Ghafari M, Nadi T, Bahadivand-Chegini S, Doosti-Irani A. Global prevalence of unmet need for mental health care among adolescents: A systematic review and meta-analysis. Arch Psychiatr Nurs. 2022 Feb;36:1-6.	Global and regional prevalence of children with mental disorders not reported
**11**	Pye K, Jackson H, Iacono T, Shiell A. Early intervention for young children with autism spectrum disorder: protocol for a scoping review of economic evaluations. Syst Rev. 2021 Nov 10;10(1):295.	Global and regional prevalence of children with ASD not reported

12	de Sena Oliveira AC, Athanasio BDS, Mrad FCC, et al. Attention deficit and hyperactivity disorder and nocturnal enuresis co-occurrence in the pediatric population: a systematic review and meta-analysis. Pediatr Nephrol. 2021 Nov;36(11):3547-3559.	Global and regional prevalence of children with ADHD not reported
13	Uchida M, Driscoll H, DiSalvo M, et al. Assessing the Magnitude of Risk for ADHD in Offspring of Parents with ADHD: A Systematic Literature Review and Meta-Analysis. J Atten Disord. 2021 Nov;25(13):1943-1948. doi: 10.1177/1087054720950815.	Global and regional prevalence of children with ADHD not reported
14	Bougeard C, Picarel-Blanchot F, Schmid R, Campbell R, Buitelaar J. Prevalence of Autism Spectrum Disorder and Co-morbidities in Children and Adolescents: A Systematic Literature Review. Front Psychiatry. 2021 Oct 27;12:744709. doi: 10.3389/fpsyt.2021.744709.	Global and regional prevalence of all children with ASD not reported
15	Ma L, Mazidi M, Li K, et al. Prevalence of mental health problems among children and adolescents during the COVID-19 pandemic: A systematic review and meta-analysis. J Affect Disord. 2021 Oct 1;293:78-89. doi: 10.1016/j.jad.2021.06.021.	Global and regional prevalence of children with mental disorders not reported
16	Jullien S. Vision screening in newborns and early childhood. BMC Pediatr. 2021 Sep 8;21(Suppl 1):306. doi: 10.1186/s12887-021-02606-2.	Global and regional prevalence of children with vision loss not reported
17	Chan MF, Al Balushi R, Al Falahi M, Mahadevan S, Al Saadoon M, Al-Adawi S. Child and adolescent mental health disorders in the GCC: A systematic review and meta-analysis. Int J Pediatr Adolesc Med. 2021 Sep;8(3):134-145. doi: 10.1016/j.ijpam.2021.04.002.	Global and regional prevalence of children with mental disorders not reported
18	Rong Y, Yang CJ, Jin Y, Wang Y. Prevalence of attention-deficit/hyperactivity disorder in individuals with autism spectrum disorder: A meta-analysis, Research in Autism Spectrum Disorders. 2021; 83: 101759	Review limited to prevalence of ADHD in children with ASD
19	Nourredine M, Gering A, Fourneret P, et al. Association of Attention-Deficit/Hyperactivity Disorder in Childhood and Adolescence with the Risk of Subsequent Psychotic Disorder: A Systematic Review and Meta-analysis. JAMA Psychiatry. 2021 May 1;78(5):519-529. doi: 10.1001/jamapsychiatry.2020.4799.	Global and regional prevalence of children with ADHD not reported
20	Sandstrom A, Perroud N, Alda M, Uher R, Pavlova B. Prevalence of attention-deficit/hyperactivity disorder in people with mood disorders: A systematic review and meta-analysis. Acta Psychiatr Scand. 2021 May;143(5):380-391. doi: 10.1111/acps.13283.	Review limited to prevalence of ADHD in children with mood disorders
21	Knight LMJ, King AA, Strouse JJ, Tanabe P. Pediatric Neurodevelopmental Delays in Children 0 to 5 Years of Age With Sickle Cell Disease: A Systematic Literature Review. J Pediatr Hematol Oncol. 2021 Apr 1;43(3):104-111. doi: 10.1097/MPH.0000000000002091.	Review limited to prevalence of developmental delays in children with sickle cell disease
22	Chhibber A, Watanabe AH, Chaisai C, Veettil SK, Chaiyakunapruk N. Global Economic Burden of Attention-Deficit/Hyperactivity Disorder: A Systematic Review. Pharmacoeconomics. 2021 Apr;39(4):399-420. doi: 10.1007/s40273-020-00998-0.	Global and regional prevalence of children with ADHD not reported
23	Lovett BJ, Nelson JM. Systematic Review: Educational Accommodations for Children and Adolescents With Attention-	Global and regional prevalence of children with ADHD not reported

	Deficit/Hyperactivity Disorder. J Am Acad Child Adolesc Psychiatry. 2021 Apr;60(4):448-457. doi: 10.1016/j.jaac.2020.07.891.	
**24**	Pan PY, Bölte S, Kaur P, Jamil S, Jonsson U. Neurological disorders in autism: A systematic review and meta-analysis. Autism. 2021 Apr;25(3):812-830. doi: 10.1177/1362361320951370.	Global and regional prevalence of children with ASD not reported
**25**	Tan C, Frewer V, Cox G, Williams K, Ure A. Prevalence and Age of Onset of Regression in Children with Autism Spectrum Disorder: A Systematic Review and Meta-analytical Update. Autism Res. 2021 Mar;14(3):582-598. doi: 10.1002/aur.2463.	Global and regional prevalence of children with ASD not reported
**26**	Di Lorenzo R, Balducci J, Poppi C, et al. Children and adolescents with ADHD followed up to adulthood: a systematic review of long-term outcomes. Acta Neuropsychiatr. 2021 Dec;33(6):283-298. doi: 10.1017/neu.2021.23.	Global and regional prevalence of children with ADHD not reported
**27**	Sarda SP, Sarri G, Siffel C. Global prevalence of long-term neurodevelopmental impairment following extremely preterm birth: a systematic literature review. J Int Med Res. 2021 Jul;49(7):3000605211028026. doi: 10.1177/03000605211028026.	Global and regional prevalence of children with disabilities not reported
**28**	Bernardo P, Cinalli G, Santoro C. Epilepsy in NF1: a systematic review of the literature. Childs Nerv Syst. 2020 Oct;36(10):2333-2350. doi: 10.1007/s00381-020-04710-7.	Global and regional prevalence of children with epilepsy not reported
**29**	Buckley N, Glasson EJ, Chen W, et al. Prevalence estimates of mental health problems in children and adolescents with intellectual disability: A systematic review and meta-analysis. Aust N Z J Psychiatry. 2020 Oct;54(10):970-984. doi: 10.1177/0004867420924101.	Global and regional prevalence of children with intellectual disability not reported
**30**	Hollingdale J, Woodhouse E, Young S, Fridman A, Mandy W. Autistic spectrum disorder symptoms in children and adolescents with attention-deficit/hyperactivity disorder: a meta-analytical review. Psychol Med. 2020 Oct;50(13):2240-2253. doi: 10.1017/S0033291719002368.	Review limited to prevalence of ASD in children with ADHD
**31**	Olusanya BO, Wright SM, Nair MKC et al; Global Research on Developmental Disabilities Collaborators (GRDDC). Global Burden of Childhood Epilepsy, Intellectual Disability, and Sensory Impairments. Pediatrics. 2020 Jul;146(1):e20192623. doi: 10.1542/peds.2019-2623.	Prevalence estimates derived from statistical modelling
**32**	Charach A, Mohammadzadeh F, Belanger SA, et al. Identification of Preschool Children with Mental Health Problems in Primary Care: Systematic Review and Meta-analysis. J Can Acad Child Adolesc Psychiatry. 2020 May;29(2):76-105.	Global and regional prevalence of children with disabilities not reported
**33**	Chiarotti F, Venerosi A. Epidemiology of Autism Spectrum Disorders: A Review of Worldwide Prevalence Estimates Since 2014. Brain Sci. 2020 May 1;10(5):274. doi: 10.3390/brainsci10050274.	This is a narrative review of studies reporting prevalence estimates
**34**	Mubashir S, Farrugia M, Coretti L, Pessia M, D'Adamo M. Autism Spectrum Disorder. Malta Medical Journal 2020;32:56-66.	Global and regional prevalence of children with ASD not reported
**35**	Butcher E, Dezateux C, Cortina-Borja M, Knowles RL. Prevalence of permanent childhood hearing loss detected at the universal newborn hearing screen: Systematic review and	Global and regional prevalence of hearing loss limited to neonates

	meta-analysis. PLoS One. 2019 Jul 11;14(7):e0219600. doi: 10.1371/journal.pone.0219600.	
**36**	Craig F, Savino R, Trabacca A. A systematic review of comorbidity between cerebral palsy, autism spectrum disorders and Attention Deficit Hyperactivity Disorder. Eur J Paediatr Neurol. 2019 Jan;23(1):31-42. doi: 10.1016/j.ejpn.2018.10.005.	Global and regional prevalence of children with disabilities not reported
**37**	Nunes ADDS, Silva CRL, Balen SA, Souza DLB, Barbosa IR. Prevalence of hearing impairment and associated factors in school-aged children and adolescents: a systematic review. Braz J Otorhinolaryngol. 2019 Mar-Apr;85(2):244-253. doi: 10.1016/j.bjorl.2018.10.009.	Global and regional prevalence of children with hearing loss not reported
**38**	Bélanger SA, Andrews D, Gray C, Korczak D. ADHD in children and youth: Part 1-Etiology, diagnosis, and comorbidity. Paediatr Child Health. 2018 Nov;23(7):447-453. doi: 10.1093/pch/pxy109.	Global and regional prevalence of children with ADHD not reported
**39**	Global Research on Developmental Disabilities Collaborators. Developmental disabilities among children younger than 5 years in 195 countries and territories, 1990-2016: a systematic analysis for the Global Burden of Disease Study 2016. Lancet Glob Health. 2018 Oct;6(10):e1100-e1121. doi: 10.1016/S2214-109X(18)30309-7	Prevalence estimates limited to young children
**40**	Foreman J, Keel S, van Wijngaarden P, et al. Prevalence and Causes of Visual Loss Among the Indigenous Peoples of the World: A Systematic Review. JAMA Ophthalmol. 2018 May 1;136(5):567-580. doi: 10.1001/jamaophthalmol.2018.0597.	Global and regional prevalence of children with vision loss not reported
**41**	Sayal K, Prasad V, Daley D, Ford T, Coghill D. ADHD in children and young people: prevalence, care pathways, and service provision. Lancet Psychiatry. 2018 Feb;5(2):175-186. doi: 10.1016/S2215-0366(17)30167-0.	This is a narrative review of studies reporting prevalence estimates of ADHD
**42**	Downs J, Blackmore AM, Epstein A, et al; Cerebral Palsy Mental Health Group. The prevalence of mental health disorders and symptoms in children and adolescents with cerebral palsy: a systematic review and meta-analysis. Dev Med Child Neurol. 2018 Jan;60(1):30-38. doi: 10.1111/dmcn.13555.	Review limited to prevalence of mental disorders in children with cerebral palsy
**43**	Erskine HE, Baxter AJ, Patton G, Moffitt TE, Patel V, Whiteford HA, Scott JG. The global coverage of prevalence data for mental disorders in children and adolescents. Epidemiol Psychiatr Sci. 2017 Aug;26(4):395-402. doi: 10.1017/S2045796015001158.	Prevalence estimates derived from statistical modelling
**44**	Bronsard G, Alessandrini M, Fond G, Loundou A, Auquier P, Tordjman S, Boyer L. The Prevalence of Mental Disorders Among Children and Adolescents in the Child Welfare System: A Systematic Review and Meta-Analysis. Medicine (Baltimore). 2016 Feb;95(7):e2622. doi: 10.1097/MD.0000000000002622.	Global and regional prevalence of children with mental disorders not reported

ASD: Autism spectrum disorder, ADHD: Attention-deficit/hyperactivity disorder
