# Supplementary material for: Global prevalence of developmental disabilities in children and adolescents: A systematic umbrella review
Source: Front Public Health. 2023 Feb 16;11:1122009. doi: 10.3389/fpubh.2023.1122009 (PMC9987263; doi:10.3389/fpubh.2023.1122009)
Supplement: Supplementary file 2 [file Data_Sheet_2.PDF]

Supplementary Table S2:Summary of findings from the quality assessment of selected studies using AMSTAR2

Authors, Year of publication [Reference]	Condition	Q1	Q2	Q3	Q4	Q5	Q6	Q7	Q8	Q9	Q10	Q11	Q12	Q13	Q14	Q15	Q16	Overall Rating
**Barican JL, et al., 2022 [23]**	ADHD	Partial Yes	Yes	Yes	Yes	Yes	Yes	Yes	Yes	Yes	No	Yes	No	No	Yes	Yes	Yes	Low
**Zeidan J., et al, 2022 [11]**	ASD	Partial Yes	No	No	No	Yes	Yes	Yes	Yes	No	No	NMA	NA	No	No	No	Yes	Critically low
**Salari N., et al, 2022 [24]**	ASD	Partial Yes	No	Yes	Yes	Yes	Yes	No	Yes	Yes	No	Yes	Yes	Yes	Yes	No	Yes	Critically low
**Wang J., et al, 2022 [25]**	ASD	Partial Yes	No	No	Yes	Yes	Yes	Yes	Yes	Yes	No	Yes	No	No	No	No	Yes	Critically low
**McIntyre S, et al, 2022 [10]**	Cerebral Palsy	Partial Yes	No	Yes	Yes	Yes	Yes	No	Yes	Yes	No	Yes	Yes	Yes	Yes	No	Yes	Critically low
**McKenzie K., et al, 2016 [26]**	Intellectual Disability	Partial Yes	No	Yes	Yes	Yes	Yes	Yes	Yes	No	No	NMA	NA	No	No	No	No	Critically low
**Fiest KM., et al, 2017 [27]**	Epilepsy	Partial Yes	Yes	Yes	Yes	Yes	Yes	Yes	Yes	Yes	No	Yes	Yes	Yes	Yes	Yes	Yes	Moderate
**Wang J., et al, 2022 [28]**	Hearing Loss	Partial Yes	Yes	Yes	Yes	Yes	Yes	Yes	Yes	Yes	No	Yes	Yes	Yes	Yes	Yes	Yes	Moderate
**Yekta A., et al, 2022 [12]**	Vision Loss	Partial Yes	No	Yes	Yes	Yes	Yes	No	Yes	Yes	No	Yes	Yes	Yes	Yes	No	Yes	Critically low
**Yang L., et al, 2022 [29]**	Dyslexia	Partial Yes	Yes	Yes	Yes	Yes	Yes	Yes	Yes	Yes	No	Yes	Yes	Yes	Yes	Yes	Yes	Moderate

ADHD: Attention-deficit/hyperactivity disorder, ASD: Autism spectrum disorder, NMA: No Meta-Analysis

AMSTAR@ Questions: **Q1:** Did the research questions and inclusion criteria for the review include the components of PICO?; **Q2:** Did the report of the review contain an explicit statement that the review methods were established prior to the conduct of the review and did the report justify any significant deviations from the protocol?; **Q3:** Did the review authors explain their selection of the study designs for inclusion in the review?; **Q4:** Did the review authors use a comprehensive literature search strategy?; **Q5:** Did the review authors perform study selection in duplicate?; **Q6:** Did the review authors perform data extraction in duplicate?; **Q7**: Did the review authors provide a list of excluded studies and justify the exclusions, *or reflected the excluded studies in PRISMA flow chart with reasons for exclusion*?; **Q8:** Did the review authors describe the included studies in adequate detail?; **Q9:** Did the review authors use a satisfactory technique for assessing the risk of bias (RoB) in individual studies that were included in the review?; **Q10:** Did the review authors report on the sources of funding for the studies included in the review?; **Q11:** If meta-analysis was performed did the review authors use appropriate methods for statistical combination of results?; **Q12:** If meta-analysis was performed, did the review authors assess the potential impact of RoB in individual studies on the results of the meta-analysis or other evidence synthesis?; **Q13:** Did the review authors account for RoB in individual studies when interpreting/ discussing the results of the review?; **Q14:** Did the review authors provide a satisfactory explanation for, and discussion of, any heterogeneity observed in the results of the review?; **Q15:** If they performed quantitative synthesis did the review authors carry out an adequate investigation of publication bias (small study bias) and discuss its likely impact on the results of the review?; **Q16:** Did the review authors report any potential sources of conflict of interest, including any funding they received for conducting the review?
