# Supplementary material for: Global prevalence of developmental disabilities in children and adolescents: A systematic umbrella review
Source: Front Public Health. 2023 Feb 16;11:1122009. doi: 10.3389/fpubh.2023.1122009 (PMC9987263; doi:10.3389/fpubh.2023.1122009)
Supplement: Supplementary file 3 [file Data_Sheet_3.PDF]

Supplementary Table S3: Summary of findings from the quality assessment of selected studies using JBI Checklist

Authors and year of publication [reference]	Condition	Q1	Q2	Q3	Q4	Q5	Q6	Q7	Q8	Q9	Q10	Overall Rating
**Barican JL, et al., 2022 [23]**	**ADHD**	Yes	Yes	Yes	Yes	Yes	Yes	Yes	Yes	Yes	Yes	10 (High)
**Zeidan J., et al, 2022 [11]**	**ASD**	Yes	Yes	Yes	No	No	No	Yes	No	Yes	Yes	6 (Medium)
**Salari N., et al, 2022 [24]**	**ASD**	Yes	Yes	Yes	Yes	Yes	No	Yes	Yes	Yes	Yes	9 (High)
**Wang J., et al, 2022 [25]**	**ASD**	Yes	Yes	Yes	Yes	Yes	Yes	Yes	Yes	Yes	Yes	10 (High)
**McIntyre S, et al, 2022 [10]**	**Cerebral Palsy**	Yes	Yes	Yes	Yes	Yes	Yes	Yes	No	Yes	Yes	9 (High)
**McKenzie K., et al, 2016 [26]**	**Intellectual Disability**	Yes	Yes	Yes	Yes	No	No	No	No	Yes	No	5 (Medium)
**Fiest KM., et al, 2017 [27]**	**Epilepsy**	Yes	Yes	Yes	Yes	Yes	Yes	Yes	Yes	Yes	Yes	10 (High)
**Wang J., et al, 2022 [28]**	**Hearing Loss**	Yes	Yes	Yes	Yes	Yes	Yes	Yes	Yes	Yes	Yes	10 (High)
**Yekta A., et al, 2022 [12]**	**Vision Loss**	Yes	Yes	Yes	Yes	Yes	Yes	Yes	No	Yes	Yes	9 (High)
**Yang L., et al, 2022 [29]**	**Dyslexia**	Yes	Yes	Yes	Yes	Yes	Yes	Yes	Yes	Yes	Yes	10 (High)

ADHD: Attention-deficit/hyperactivity disorder, ASD: Autism spectrum disorder

JBI Questions: **Q1**: Is the review question clearly and explicitly stated? **Q2**: Were the inclusion criteria appropriate for the review question? **Q3:** Was the search strategy appropriate? **Q4** Were the sources and resources used to search for studies adequate? **Q5:** Were the criteria for appraising studies appropriate? **Q6:** Was critical appraisal conducted by two or more reviewers independently? **Q7:** Were the methods used to combine studies appropriate? **Q8:** Was the likelihood of publication bias assessed? **Q9:** Were recommendations for policy and/or practice supported by the reported data? **Q10:** Were the specific directives for new research appropriate?
